# Safety of Sarilumab in the treatment of rheumatoid arthritis: a real-world study based on the FAERS database

**DOI:** 10.3389/fmed.2025.1665293

**Published:** 2025-09-08

**Authors:** Dahui Wang, Qian Guo, Xin Chen, Jinguang He, Jiehua Deng

**Affiliations:** ^1^Department of Plastic and Aesthetic Surgery, The Second Affiliated Hospital of Guilin Medical University, Guilin, China; ^2^Neurobiology of Pain Research Group, School of Medicine, Institute of Neurosciences, University of Barcelona, Barcelona, Spain; ^3^Department of Rhinology, The First Affiliated Hospital of Zhengzhou University, Zhengzhou, China; ^4^Department of Anesthesiology, The Second Affiliated Hospital of Hainan Medical University, Haikou, China; ^5^Department of Anesthesiology, Affiliated Nanhua Hospital, University of South China, Hengyang, China

**Keywords:** Sarilumab, rheumatoid arthritis, FAERS database, adverse drug events, real-world study

## Abstract

**Background:**

Rheumatoid Arthritis (RA) is a chronic autoimmune disease causing joint pain, swelling, and systemic symptoms like fatigue, with higher incidence in females and increased risk with age. It often co-exists with depression, imposing a significant global health burden with high annual cases and substantial economic losses. Sarilumab, a fully humanized IgG1 monoclonal antibody targeting the IL-6 receptor α, has shown efficacy in clinical trials but requires real-world safety monitoring.

**Methods:**

This study analyzed Sarilumab-related adverse drug event (ADE) reports from the FAERS database (Q2 2017–Q1 2025). Data were cleaned and analyzed using SAS 9.4, with signal detection via Reporting Odds Ratio (ROR), Proportional Reporting Ratio (PRR), Bayesian Confidence Propagation Neural Network (BCPNN), and Multivariate Gamma-Poisson Shrinker (MGPS).

**Results:**

A total of 14,940 Sarilumab users with 42,730 adverse events (AEs) were included. AE signals were detected across all 26 System Organ Classes (SOC), with the highest in Infections and Infestations (62 signals), General Disorders and Administration Site Conditions (44), Musculoskeletal and Connective Tissue Disorders (36), and Surgical and Medical Procedures (36). Nearly half of AEs occurred within 30 days (49.56%), and 15.79% after 360 days. Common AEs included pain, joint swelling, and injection site reactions, while severe reactions included serious infections and anaphylaxis. Some AEs not listed on the drug label, such as fatigue and alopecia, were also identified.

**Conclusion:**

This real-world analysis highlights Sarilumab’s safety profile, emphasizing the need for close patient monitoring, particularly during the early initiation phase and throughout long-term treatment. The findings provide valuable insights for clinicians in pre-medication assessment, during-treatment monitoring, and adverse reaction management, while underscoring the need for further research into the mechanisms and risk factors of these adverse events.

## 1 Introduction

Rheumatoid Arthritis (RA) is a chronic, systemic autoimmune disease, characterized clinically by symmetrical arthritis, joint pain, swelling, and limited motor function (1). In addition to joint damage, patients with RA often present with systemic symptoms such as fatigue, fever, and weight loss. In severe cases, joint deformity and loss of function may occur, significantly reducing the patients’ quality of life (2). According to an analysis based on the publicly available data from the 2019 Global Burden of Disease (GBD) Study, the global incidence of RA is approximately 0.5–1%. The incidence is higher in females than in males, and the risk of developing the disease increases with age (3). Despite certain advancements in understanding the pathogenesis and formulating treatment strategies for rheumatoid arthritis (RA), the disease remains a significant global public health concern (2), continuously imposing a heavy burden on individual health and the healthcare system. Statistics show that RA causes approximately 1.8 million cases annually, resulting in a global economic loss of around 326 billion US dollars (4).

Interleukin-6 (IL-6) is a key cytokine mediating multiple inflammatory responses, and its expression is significantly elevated in the serum and joint synovial fluid of RA patients, making it an important therapeutic target for RA (5). Sarilumab is a fully humanized IgG1 subclass monoclonal antibody that targets the IL-6 receptor α (including soluble sIL-6Rα and membrane-bound mIL-6Rα), effectively blocking IL-6-mediated signal transduction and regulating inflammation (6). Based on this mechanism, Sarilumab was approved by the U.S. FDA in 2017 for the treatment of adult patients with moderately to severely active RA who have had an inadequate response or intolerance to one or more traditional disease-modifying antirheumatic drugs (DMARDs) (7).

Although Sarilumab has demonstrated promising efficacy in clinical trials, post-marketing real-world adverse events require continuous monitoring. The U.S. FDA Adverse Event Reporting System (FAERS) (8), an open pharmacovigilance database, collects adverse reaction reports from clinical use, providing critical data support for drug safety assessments and enabling the identification of potential risks not recognized in preclinical studies.

This study systematically retrieved Sarilumab-related adverse drug event (ADE) reports from the FAERS database and used multiple signal detection methods for quantitative analysis across multiple dimensions, aiming to identify potential safety signals, alert clinicians to possible risks, and improve medication safety for RA patients.

## 2 Methods

### 2.1 Data source

The data used in this study were obtained from the FAERS database publicly released on the official website of the U.S. Food and Drug Administration (FDA). The database has been open to the public since Q1 2004 and is updated quarterly, offering data packages in two formats (ASCII and XML) for researchers to download. This study selected the original ASCII format packages as the basis for data mining and statistical analysis. To systematically analyze Sarilumab-related ADEs, a total of 85 quarterly datasets from Q2 2017 to Q1 2025 were extracted. All data were subsequently imported into the SAS 9.4 software platform for cleaning and statistical analysis.

### 2.2 Data management

Data management involved duplicate report removal and AE terminology standardization. First, duplicate reports were deleted using FDA-recommended methods. Specifically, reports were categorized by case identifier (CASEID), FDA receipt date (FDA_DT), and each report’s unique identifier (PRIMARYID). For reports with the same CASEID, the one with the most recent FDA_DT was retained; if CASEID and FDA_DT were identical, the report with the largest PRIMARYID was kept. Second, starting from Q1 2019, duplicate data were removed based on CASEID values in the quarterly deletion report lists. Third, the latest version of the Medical Dictionary for Regulatory Activities (MedDRA 27.1) was used to code AE terms in the FAERS database, including preferred terms (PT) and corresponding system organ class (SOC) classifications, which were used for subsequent analyses.

### 2.3 Signal detection methods

This study employed disproportionality methods including the Reporting Odds Ratio (ROR), Proportional Reporting Ratio (PRR), Bayesian Confidence Propagation Neural Network (BCPNN), and Multivariate Gamma-Poisson Shrinker (MGPS) for ADE signal detection. The PRR method used the comprehensive criteria recommended by the UK Medicines and Healthcare products Regulatory Agency (MHRA) as the signal detection threshold.

A potential adverse reaction was identified when an AE exceeded the positive determination threshold in at least one method. A detailed 2 × 2 contingency table is shown in Supplementary Table S1, and the formulas and threshold criteria for each disproportionality analysis method are listed in Supplementary Table S2. The occurrence time of Sarilumab-related AEs was defined as the time interval between the AE occurrence date (DEMO file) and the drug start date (THER file). To model the temporal variation in AE incidence, a Weibull distribution was used.

To balance sensitivity and specificity, detections by a single method (ROR, PRR, BCPNN, or MGPS) were regarded as exploratory and were not used alone for clinical recommendations. Specificity was supported by applying established thresholds (e.g., MHRA criteria and a minimum case count ≥3) and by interpreting detected signals in conjunction with clinical plausibility, targeted literature verification, and stratified analyses (9–11). This strategy preserves sensitivity for rare or emerging events while mitigating over-interpretation of spurious findings.

### 2.4 Stratified analysis and drug–drug interaction (DDI) assessment

To investigate potential heterogeneity in adverse event (AE) reporting patterns, stratified disproportionality analyses were performed according to sex (male, female), age group (18–44 years, 45–64 years, ≥65 years), and reporter type (healthcare professional, consumer, pharmacist). Within each stratum, four disproportionality methods—Reporting Odds Ratio (ROR), Proportional Reporting Ratio (PRR), Bayesian Confidence Propagation Neural Network (BCPNN), and Multivariate Gamma–Poisson Shrinker (MGPS)—were applied independently. A signal was considered positive when the predefined threshold was met in at least one method. This approach allowed for the identification of population-specific safety signals that might be masked in pooled analyses.

Potential drug–drug interactions (DDIs) were assessed using the *Ω* shrinkage measure model, which evaluates whether the observed frequency of a specific AE associated with a pair of drugs exceeds the expected frequency assuming independence. The *Ω* statistic was calculated as:


$$\Omega =\log_{2}\;((N_{11}+\alpha )/(E_{11}+\alpha ))$$

where *N*_11_ is the number of reports containing both drugs and the AE of interest, *E*_11_ is the expected number of such reports under the assumption of independence, and *α* is a shrinkage parameter (commonly set to 0.5) used to stabilize estimates in sparse data. The expected value E_11_ was calculated as:


$$E_{11}=(N_{1}\cdot \times N\cdot_{1})/N$$

where *N*_1_· is the number of reports containing both drugs, *N*·_1_ is the number of reports containing the AE, and *N* is the total number of reports in the dataset. The 95% confidence interval (CI) for *Ω* was obtained by:


$$\Omega \_\mathrm{CI}=\log_{2}\;((N_{11}+\alpha )/(E_{11}+\alpha ))\pm 1.96\times \mathrm{SE}(\Omega )$$

An interaction signal was considered statistically significant if the lower bound of the 95% CI exceeded zero (*Ω*_lower > 0).

Several frequency-based statistical models are available for detecting potential DDIs, including the combined risk ratio (CRR) model, reporting ratio method, *χ*^2^ statistics model, and *Ω* shrinkage measure model. The CRR model evaluates the relative risk of an AE in the presence of a drug pair compared with individual drugs, but it may be unstable when event counts are low (12). The reporting ratio method is simple and intuitive; however, it lacks adjustment for data sparsity and may produce inflated estimates for rare events (13). The *χ*^2^ statistics model assesses deviations between observed and expected counts, yet it can also be overly sensitive to small cell counts, leading to false positives (14). In contrast, the *Ω* shrinkage measure model incorporates a shrinkage parameter to stabilize estimates in sparse data, thereby reducing random fluctuation and false-positive signals, particularly for rare or emerging AEs (15) Given the low frequency of most DDI reports in FAERS and the need for robust estimation under sparse conditions, the *Ω* shrinkage measure model was selected as the primary approach in this study.

No minimum sample size threshold was imposed for DDI detection, in line with exploratory signal detection principles in FAERS analyses. However, co-report counts (*N*_11_) are presented for transparency, and low-count findings should be interpreted with caution.

### 2.5 Sensitivity analysis by indication

In addition to the primary analysis including all primary-suspect (PS) reports irrespective of indication, we performed a sensitivity analysis restricted to reports with “Rheumatoid arthritis” recorded as the indication (INDI_PT), to evaluate the robustness of the findings.

## 3 Results

### 3.1 Clinical characteristics

All signal counts reported in the following sections represent exploratory findings based on at least one method being positive; they were subsequently interpreted with consideration of clinical plausibility, literature verification, and stratified analyses to reduce the risk of false positives.

**Figure 1:**
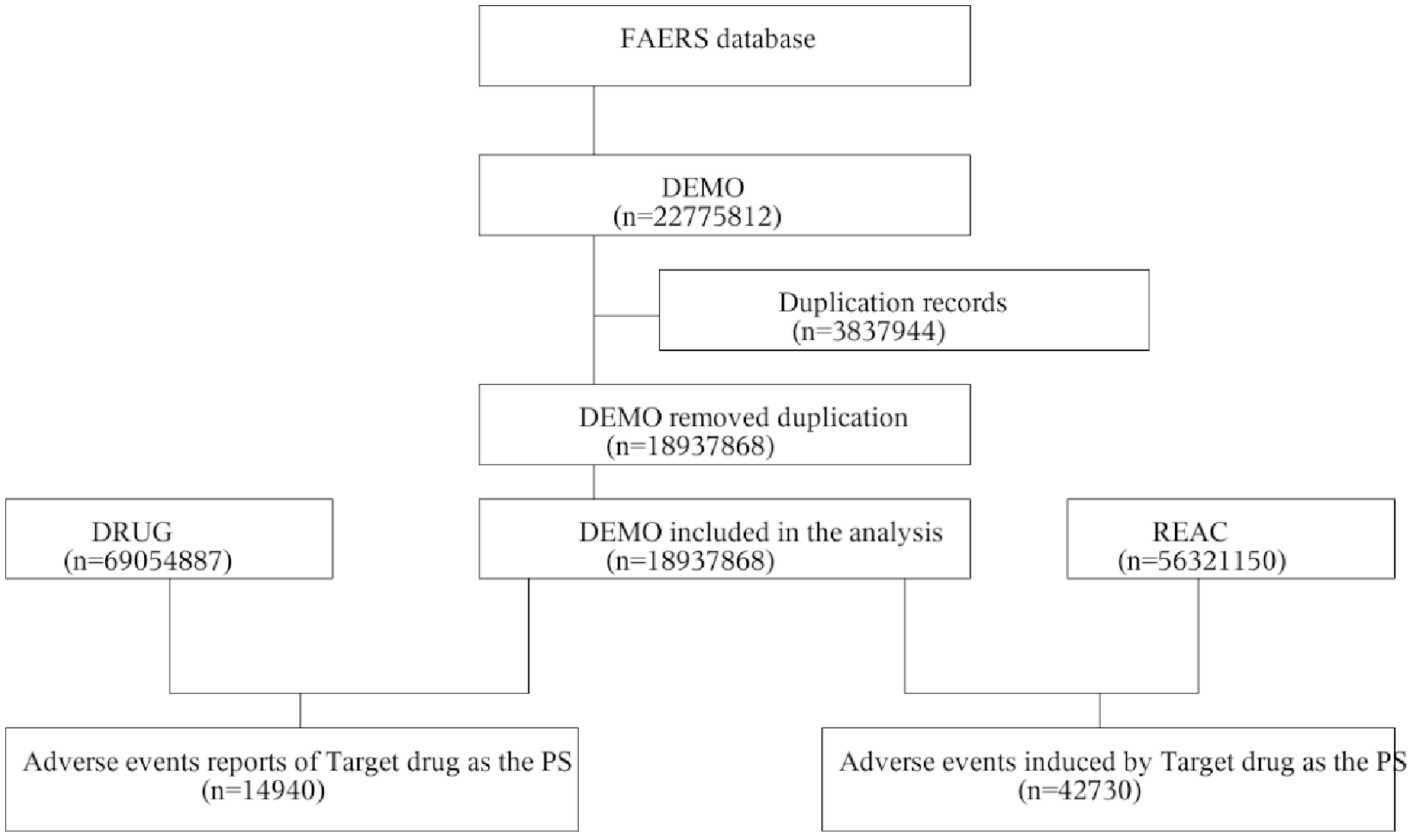
Flowchart of AE analysis for sarilumab using the FAERS database. AE, adverse event; DEMO, demographic and administrative information file; FAERS, FDA Adverse Event Reporting System; PS, Primary Suspect drug; REAC, adverse event information file.

A total of 14,940 patients using the target drug were included, with 42,730 adverse event (AE) cases recorded, where Sarilumab was identified as the primary suspected drug (Figure 1). The drug is primarily indicated for RA patients and is administered via subcutaneous injection. In terms of patient characteristics, females accounted for 75.60%, males 15.90%. The 45–64 age group had the highest proportion (37.07%), followed by the ≥65 age group (20.00%). Most reports were submitted by physicians (54.23%), followed by consumers (37.59%) and pharmacists (6.76%). Geographically, reports were predominantly from North America (93.0%), with the U.S. accounting for 91.62%. In terms of severity, non-serious AEs accounted for 72.60%, and serious AEs 27.40%. Temporally, AEs primarily occurred between 2017 and 2025, showing an annual upward trend and peaking in 2024 (3,230 cases). Additionally, 6.47% of AEs occurred within 30 days of medication. More details are shown in Table 1.

**Table 1:** Clinical characteristics of sarilumab-related AE reports in FAERS database (Q2 2017–Q1 2025).

Characteristics	*n* (%)
Sex	
Female	11,294 (75.60)
Male	2,376 (15.90)
Not specified	1,270 (8.50)
Age (years)	
<18	14 (0.09)
18–44	1,265 (8.47)
45–64	5,539 (37.07)
≥65	2,988 (20.00)
Not specified	5,134 (34.36)
Age (years)	
*N* (missing)	9,806 (5134)
Mean (SD)	58.76 (12.59)
Median (Q1, Q3)	59.00 (52.00, 67.00)
Min, Max	0.16, 97.00
Report year	
2017	96 (0.64)
2018	909 (6.08)
2019	1,595 (10.68)
2020	1,510 (10.11)
2021	1,896 (12.69)
2022	2,316 (15.50)
2023	2,194 (14.69)
2024	3,230 (21.62)
2025	1,194 (7.99)
Reporter	
Consumer	5,616 (37.59)
Lawyer	1 (0.01)
Not specified	15 (0.10)
Other health-professional	196 (1.31)
Pharmacist	1,010 (6.76)
Physician	8,102 (54.23)
Outcomes	
Life-threatening	185 (1.24)
Hospitalization—initial or prolonged	1,309 (8.76)
Disability	431 (2.88)
Death	260 (1.74)
Congenital anomaly	26 (0.17)
Required intervention to prevent permanent impairment/damage	5 (0.03)
Other	3,307 (22.14)
Adverse event occurrence time (days)	
0–30 days	967 (6.47)
31–60 days	168 (1.12)
61–90 days	108 (0.72)
91–120 days	83 (0.56)
121–150 days	68 (0.46)
151–180 days	54 (0.36)
181–360 days	195 (1.31)
>360 days	308 (2.06)
Missing or outlier (less than 0) (%)	12,989 (86.94)
Adverse event occurrence time (days)	
*N* (missing)	1951 (12989)
Mean (SD)	187.98 (352.19)
Median (Q1, Q3)	31.00 (1.00, 192.00)
Min, Max	0.00, 3917.00
Weight (kg)	
*N* (missing)	1,449 (13491)
Mean (SD)	82.90 (27.14)
Median (Q1, Q3)	78.50 (65.45, 97.00)
Min, Max	1.00, 273.00

### 3.2 AE distribution at SOC level

**Figure 2:**
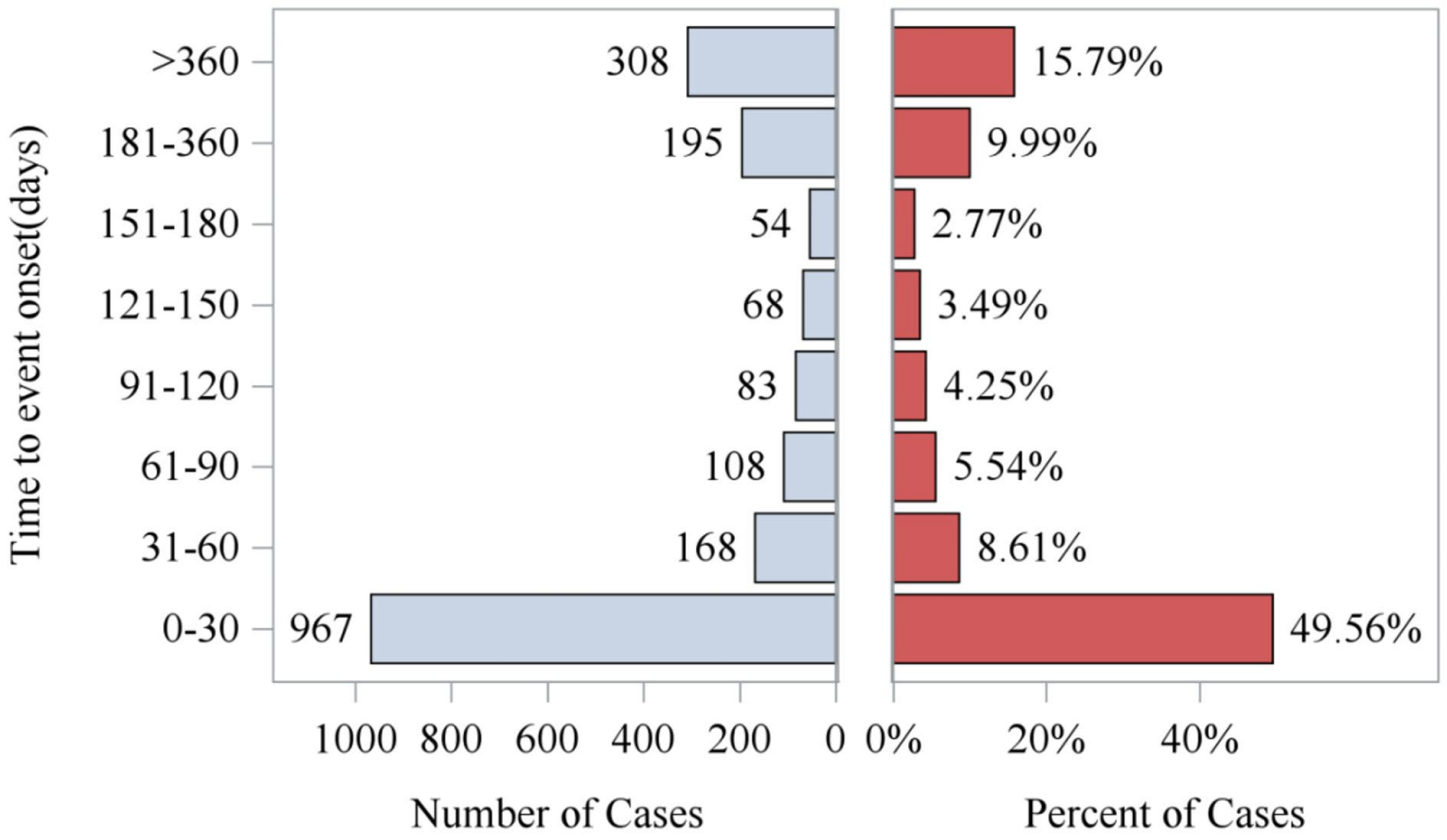
Time to event report distribution of AE reports. AE, adverse event.

Analysis of the FAERS database showed that Sarilumab-related AEs had signals detected in all 26 System Organ Classes (SOC). Among these, 11 SOCs had at least 10 signals detected by any algorithm, as shown in Table 2. The highest number of signals were in Infections and Infestations (62), General Disorders and Administration Site Conditions (44), Musculoskeletal and Connective Tissue Disorders (36), and Surgical and Medical Procedures (36). These systems are key safety focus areas for this drug and require clinical attention (Table 2). Additionally, AEs within 0–30 days of medication accounted for the highest proportion (49.56%) across all time periods, indicating a clear early-onset risk signal. Notably, 15.79% of AEs occurred more than 360 days after medication, suggesting potential long-term risks of the drug and emphasizing the need for enhanced long-term follow-up (Figure 2).

**Table 2:** Signal strength of ADEs at the System Organ Class (SOC) level in FAERS database.

System Organ Class (SOC)	SOC code	Case reports	ROR (95% CI)	PRR (95% CI)	Chi_ square	IC (IC025)	EBGM (EBGM05)
General disorders and administration site conditions	10,018,065	13,375	2.16 (2.11, 2.2)	1.80 (1.77, 1.82)	5707.17	0.84 (0.82)	1.79 (1.76)
Musculoskeletal and connective tissue disorders	10,028,395	6,719	3.43 (3.34, 3.5)	3.05 (2.98, 3.12)	9739.43	1.61 (1.57)	3.05 (2.97)
Injury, poisoning and procedural complications	10,022,117	3,809	0.84 (0.81, 0.87)	0.85 (0.83, 0.88)	109.43	−0.23 (−0.28)	0.85 (0.82)
Infections and infestations	10,021,881	3,297	1.50 (1.45, 1.56)	1.47 (1.42, 1.51)	514.96	0.55 (0.50)	1.47 (1.41)
Skin and subcutaneous tissue disorders	10,040,785	2,722	1.19 (1.14, 1.24)	1.18 (1.14, 1.22)	77.10	0.24 (0.18)	1.18 (1.13)
Gastrointestinal disorders	10,017,947	2,373	0.63 (0.61, 0.66)	0.65 (0.63, 0.68)	476.45	−0.61 (−0.67)	0.65 (0.63)
Investigations	10,022,891	1868	0.70 (0.67, 0.73)	0.71 (0.68, 0.75)	227.92	−0.49(−0.55)	0.71(0.68)
Respiratory, thoracic and mediastinal disorders	10,038,738	1,641	0.81 (0.77, 0.85)	0.82 (0.78, 0.86)	71.38	−0.29(−0.37)	0.82(0.78)
Nervous system disorders	10,029,205	1,635	0.43 (0.41, 0.45)	0.45 (0.43, 0.47)	1183.75	−1.14 (−1.22)	0.45 (0.43)
Social circumstances	10,041,244	795	4.05 (3.77, 4.34)	3.99 (3.73, 4.28)	1785.37	1.99 (1.88)	3.98 (3.71)
Surgical and medical procedures	10,042,613	742	1.28 (1.19, 1.38)	1.28 (1.19, 1.37)	45.60	0.35 (0.25)	1.28 (1.19)
Psychiatric disorders	10,037,175	688	0.28 (0.26, 0.30)	0.29 (0.27, 0.31)	1290.55	−1.80 (−1.91)	0.29 (0.27)
Immune system disorders	10,021,428	530	1.13 (1.03, 1.23)	1.12 (1.03, 1.22)	7.34	0.17 (0.04)	1.12 (1.03)
Vascular disorders	10,047,065	339	0.37 (0.33, 0.41)	0.37 (0.34, 0.41)	365.34	−1.42 (−1.58)	0.37 (0.34)
Blood and lymphatic system disorders	10,005,329	321	0.44 (0.39, 0.49)	0.44 (0.40, 0.50)	226.20	−1.17 (−1.33)	0.44 (0.40)
Cardiac disorders	10,007,541	314	0.28 (0.25, 0.31)	0.28(0.25,0.31)	592.81	−1.83 (−1.99)	0.28 (0.25)
Metabolism and nutrition disorders	10,027,433	291	0.31 (0.28, 0.35)	0.31 (0.28, 0.35)	444.73	−1.67 (−1.83)	0.31(0.28)
Eye disorders	10,015,919	271	0.31 (0.28, 0.35)	0.32 (0.28, 0.36)	404.79	−1.65(−1.82)	0.32(0.28)
Renal and urinary disorders	10,038,359	247	0.30 (0.27, 0.34)	0.31 (0.27, 0.35)	398.50	−1.71 (−1.89)	0.31 (0.27)
Neoplasms benign, malignant and unspecified (Incl cysts and polyps)	10,029,104	243	0.21 (0.19, 0.24)	0.22 (0.19, 0.25)	699.50	−2.20 (−2.38)	0.22 (0.19)
Hepatobiliary disorders	10,019,805	179	0.45 (0.39, 0.52)	0.46 (0.39, 0.53)	117.53	−1.13 (−1.35)	0.46 (0.39)
Ear and labyrinth disorders	10,013,993	104	0.56 (0.46, 0.68)	0.56 (0.46, 0.68)	35.88	−0.83 (−1.11)	0.56 (0.46)
Product issues	10,077,536	101	0.14 (0.12, 0.17)	0.14 (0.12, 0.18)	522.00	−2.79 (−3.07)	0.14 (0.12)
Reproductive system and breast disorders	10,038,604	54	0.14 (0.11, 0.19)	0.14 (0.11, 0.19)	280.15	−2.81 (−3.17)	0.14 (0.11)
Endocrine disorders	10,014,698	29	0.27 (0.18, 0.38)	0.27 (0.18, 0.38)	58.99	−1.91 (−2.40)	0.27 (0.18)
Pregnancy, puerperium and perinatal conditions	10,036,585	28	0.15 (0.11, 0.22)	0.15 (0.11, 0.22)	131.02	−2.70 (−3.19)	0.15 (0.11)
Congenital, familial and genetic disorders	10,010,331	15	0.12(0.07, 0.19)	0.12 (0.07, 0.19)	99.93	−3.09 (−3.73)	0.12 (0.07)

Ranked by case reports.

### 3.3 AE distribution at the preferred term (PT) level

Adverse events related to Sarilumab were analyzed based on the frequency of PT occurrence, and potential safety signals were evaluated. Among the 50 most common adverse events, known common side effects included pain, joint swelling, injection site reactions, decreased white blood cell count, nausea, and increased risk of infection. Severe adverse reactions (although rare but possible) included severe infections, anaphylaxis, gastrointestinal perforation, hepatotoxicity, etc. Additionally, adverse events not listed on the drug label, such as fatigue and alopecia, were also identified. Table 3 details the 50 most common adverse events during Sarilumab treatment. Table 4 presents classic and non-classic adverse events of Sarilumab and their clinical implications.

**Table 3:** Top 50 most frequent AEs for Sarilumab at the preferred term (PT) level.

System Organ Class (SOC)	Preferred term (PT)	Case reports	ROR (95% CI)	PRR (95% CI)	Chi Square	IC (IC025)	EBGM (EBGM05)
General disorders and administration site conditions	Drug ineffective	2,244	2.56 (2.45, 2.67)	2.48 (2.38, 2.58)	2016.05	1.31 (1.24)	2.47 (2.37)
General disorders and administration site conditions	Pain	2097	5.10 (4.88, 5.33)	4.90 (4.70, 5.11)	6552.62	2.29 (2.22)	4.89 (4.68)
Musculoskeletal and connective tissue disorders	Arthralgia	1897	7.04 (6.72, 7.37)	6.77 (6.48, 7.07)	9338.03	2.75 (2.68)	6.74 (6.43)
Musculoskeletal and connective tissue disorders	Rheumatoid arthritis	1,244	17.43 (16.47, 18.44)	16.95(16.04,17.91)	18464.8	4.07 (3.96)	16.75 (15.82)
Musculoskeletal and connective tissue disorders	Joint swelling	1,241	16.01 (15.13, 16.95)	15.58 (14.74, 16.46)	16760.2	3.95 (3.85)	15.40 (14.55)
General disorders and administration site conditions	Condition aggravated	974	5.00 (4.69, 5.33)	4.91 (4.61, 5.22)	3031.61	2.29 (2.19)	4.89 (4.59)
Injury, poisoning and procedural complications	Product use in unapproved indication	816	5.29 (4.93,5.67)	5.21 (4.86,5.57)	2772.94	2.38 (2.27)	5.19 (4.84)
General disorders and administration site conditions	Injection site erythema	690	8.30 (7.70, 8.95)	8.18 (7.60, 8.81)	4331.97	3.02 (2.90)	8.14 (7.55)
Social circumstances	Loss of personal independence in daily activities	684	15.07 (13.97, 16.26)	14.84 (13.77, 16.00)	8742.41	3.88 (3.74)	14.69 (13.62)
General disorders and administration site conditions	Injection site pruritus	610	13.76 (12.69, 14.91)	13.57 (12.54, 14.69)	7039.61	3.75 (3.60)	13.44 (12.41)
General disorders and administration site conditions	Injection site swelling	478	9.57 (8.74, 10.48)	9.48 (8.67, 10.36)	3602.65	3.24 (3.08)	9.42 (8.60)
General disorders and administration site conditions	Injection site reaction	416	9.01 (8.18, 9.93)	8.93 (8.12, 9.83)	2914.89	3.15 (2.98)	8.88 (8.06)
General disorders and administration site conditions	Gait disturbance	361	2.65 (2.39, 2.94)	2.64 (2.38, 2.92)	367.01	1.40 (1.24)	2.63 (2.37)
General disorders and administration site conditions	Injection site rash	354	18.02 (16.22, 20.02)	17.88 (16.10, 19.84)	5567.01	4.14 (3.92)	17.65 (15.89)
General disorders and administration site conditions	Peripheral swelling	335	3.27 (2.94, 3.64)	3.25 (2.92, 3.62)	523.18	1.70 (1.53)	3.25 (2.92)
Infections and infestations	COVID-19	328	2.64 (2.37, 2.95)	2.63 (2.36, 2.93)	331.70	1.39 (1.23)	2.63 (2.36)
Investigations	White blood cell count decreased	315	4.21 (3.77, 4.70)	4.18 (3.75, 4.67)	762.33	2.06 (1.88)	4.17 (3.74)
General disorders and administration site conditions	Illness	304	5.13 (4.59, 5.75)	5.11 (4.56, 5.71)	1001.14	2.35 (2.16)	5.09 (4.55)
Infections and infestations	Infection	298	3.09 (2.76, 3.46)	3.08 (2.75, 3.44)	417.29	1.62 (1.44)	3.07 (2.74)
Infections and infestations	Nasopharyngitis	298	2.37 (2.12, 2.66)	2.36 (2.11, 2.65)	234.56	1.24 (1.06)	2.36 (2.11)
General disorders and administration site conditions	Swelling	202	2.68 (2.33, 3.07)	2.67 (2.33, 3.06)	210.70	1.41 (1.20)	2.67 (2.32)
Musculoskeletal and connective tissue disorders	Musculoskeletal stiffness	180	3.00 (2.59, 3.47)	2.99 (2.58, 3.46)	237.71	1.58 (1.35)	2.98 (2.58)
Surgical and medical procedures	Surgery	178	4.80 (4.14, 5.57)	4.79 (4.13, 5.54)	531.70	2.25 (2.01)	4.77 (4.12)
Respiratory, thoracic and mediastinal disorders	Oropharyngeal pain	177	2.77 (2.39, 3.22)	2.77 (2.39, 3.21)	199.69	1.47 (1.24)	2.76 (2.38)
General disorders and administration site conditions	Injection site bruising	172	3.29 (2.83, 3.82)	3.28 (2.82, 3.80)	271.67	1.71 (1.47)	3.27 (2.82)
Infections and infestations	Sinusitis	171	2.42 (2.08, 2.81)	2.41 (2.08, 2.80)	141.41	1.27 (1.04)	2.41 (2.07)
Respiratory, thoracic and mediastinal disorders	Rhinorrhoea	167	3.83 (3.29, 4.46)	3.82 (3.29, 4.45)	347.46	1.93 (1.68)	3.81 (3.28)
General disorders and administration site conditions	Therapeutic response decreased	167	4.10 (3.52, 4.78)	4.09 (3.52, 4.76)	389.27	2.03 (1.78)	4.08 (3.51)
Investigations	Hepatic enzyme increased	149	3.33 (2.84, 3.91)	3.32 (2.83, 3.90)	241.76	1.73 (1.47)	3.32 (2.82)
Injury, poisoning and procedural complications	Product dose omission in error	127	8.95 (7.51, 10.66)	8.92 (7.50, 10.62)	887.96	3.15 (2.81)	8.87 (7.45)
Musculoskeletal and connective tissue disorders	Mobility decreased	118	2.43 (2.03, 2.92)	2.43 (2.03, 2.91)	99.26	1.28 (1.00)	2.43 (2.03)
Respiratory, thoracic and mediastinal disorders	Nasal congestion	116	2.94 (2.45, 3.53)	2.94 (2.45, 3.53)	148.16	1.55 (1.26)	2.93 (2.45)
General disorders and administration site conditions	Gait inability	112	2.81 (2.33, 3.38)	2.80 (2.33, 3.37)	129.51	1.48 (1.19)	2.80 (2.32)
General disorders and administration site conditions	Injection site urticaria	112	7.00 (5.81, 8.43)	6.98 (5.80, 8.40)	571.23	2.80 (2.45)	6.95 (5.77)
General disorders and administration site conditions	Inflammation	112	3.27 (2.71, 3.93)	3.26 (2.71, 3.93)	175.38	1.70 (1.40)	3.26 (2.70)
Investigations	Blood cholesterol increased	109	3.42 (2.83, 4.13)	3.41 (2.83, 4.12)	185.45	1.77 (1.46)	3.41 (2.82)
Gastrointestinal disorders	Stomatitis	108	2.62 (2.17, 3.17)	2.62 (2.17, 3.16)	107.91	1.39 (1.09)	2.62 (2.16)
General disorders and administration site conditions	Injection site mass	91	3.64 (2.96, 4.47)	3.63 (2.96, 4.46)	173.32	1.86 (1.52)	3.63 (2.95)
General disorders and administration site conditions	Injection site warmth	85	5.83 (4.71, 7.22)	5.82 (4.71, 7.21)	338.29	2.54 (2.15)	5.80 (4.69)
Musculoskeletal and connective tissue disorders	Joint stiffness	70	3.90 (3.08, 4.93)	3.89 (3.08, 4.92)	150.00	1.96 (1.56)	3.88 (3.07)
Musculoskeletal and connective tissue disorders	Systemic lupus erythematosus	65	3.09 (2.42, 3.94)	3.09 (2.42, 3.94)	91.53	1.62 (1.22)	3.08 (2.42)
Infections and infestations	Diverticulitis	63	3.36 (2.63, 4.31)	3.36 (2.62, 4.30)	104.17	1.75 (1.33)	3.35 (2.62)
Surgical and medical procedures	Knee arthroplasty	60	4.48 (3.48, 5.77)	4.47 (3.47, 5.76)	161.34	2.16 (1.71)	4.46 (3.46)
Injury, poisoning and procedural complications	Intentional dose omission	60	3.91 (3.03, 5.03)	3.90 (3.03, 5.03)	129.16	1.96 (1.52)	3.89 (3.02)
Musculoskeletal and connective tissue disorders	Polymyalgia rheumatica	59	30.37 (23.46, 39.32)	30.33 (23.44, 39.25)	1635.86	4.89 (3.95)	29.67 (22.92)
Infections and infestations	Ear infection	55	3.07 (2.35, 4.00)	3.07 (2.35, 3.99)	76.43	1.61 (1.18)	3.06 (2.35)
General disorders and administration site conditions	Injection site irritation	54	6.30 (4.82, 8.23)	6.29 (4.82, 8.22)	239.15	2.65 (2.13)	6.26 (4.79)
Infections and infestations	Localised infection	54	3.22 (2.46, 4.20)	3.21 (2.46, 4.19)	82.11	1.68 (1.23)	3.21 (2.45)
General disorders and administration site conditions	Therapeutic response shortened	53	2.91 (2.22, 3.81)	2.91 (2.22, 3.80)	66.13	1.54 (1.09)	2.90 (2.22)
Musculoskeletal and connective tissue disorders	Hand deformity	48	6.48 (4.88, 8.60)	6.47 (4.88, 8.59)	221.06	2.69 (2.12)	6.45 (4.85)

**Table 4:** Representative classic and non-classic AEs of Sarilumab and their clinical implications.

Adverse effect type	Classic adverse effects	Non-classic adverse effects	Clinical implications
Musculoskeletal and connective tissue disorders	- Arthralgia - Joint swelling	- Musculoskeletal stiffness	- Seek medical attention promptly to assess whether a dose adjustment or change in treatment regimen is necessary.
Administration site conditions	- Injection site erythema - Injection site pruritus	- Gait disturbance	- Manage mild swelling with site rotation and cold therapy. - Consult a doctor promptly for severe pain or systemic reactions.
Gastrointestinal	- Abdominal discomfort - Nausea	- GI perforation	- Provide symptomatic treatment - Ensure hydration
General disorders	- Pain - Drug ineffective	- Anaphylaxis	- Monitor for signs of hypersensitivity - Have emergency protocols in place.
Other	- Drug-induced hepatotoxicity	- Acute liver injury	- Monitor liver function - Stop use and consult a doctor if symptoms are severe.

In addition, cardiovascular and nervous system–related adverse events were also identified. Cardiovascular events, such as hypertension (*n* = 12), palpitations (*n* = 9), tachycardia (*n* = 7), hypotension (*n* = 5), bradycardia (*n* = 4), and QT interval prolongation (*n* = 3), corresponded to 314 cases classified as cardiac disorders and 339 cases classified as vascular disorders. Nervous system events, such as headache (*n* = 15), dizziness (*n* = 11), paraesthesia (*n* = 6), loss of consciousness (*n* = 4), seizures (*n* = 3), and memory impairment (*n* = 2), totaled 1,635 cases. Although their overall frequencies were lower than infection- or musculoskeletal-related events, several exhibited positive disproportionality signals, warranting clinical attention.

### 3.4 AE onset time

**Figure 3:**
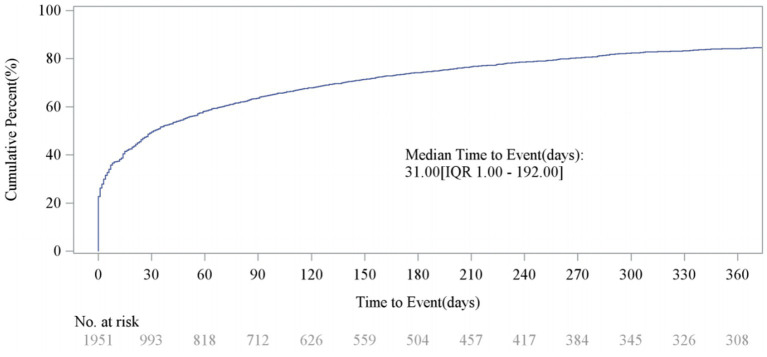
Cumulative incidence of AEs. AE, adverse event.

After excluding unreliable reports, a total of 1,951 Sarilumab-related AEs provided onset time data. Nearly half of the AEs occurred within 30 days of medication (*n* = 967, 6.47%). The median onset time was 0 days [interquartile range (IQR) 0.00–0.00 days]. The timeline of these events is shown in Figure 2. The cumulative incidence curve of AEs is shown in Figure 3. Furthermore, the Weibull distribution test in Table 5 indicates that the failure type for Sarilumab-associated AEs is consistent with early failure, with a shape parameter *β* of 0.60 (95% CI: 0.58–0.63).

**Table 5:** Time-to-onset analysis using the Weibull distribution test.

Cases	TTO (days)	Weibull distribution				
		Scale parameter		Shape parameter		
*n*	Median (IQR)	*α*	95% CI	*β*	95% CI	Failure type
1951	31.00 (1.00, 192.00)	161.14	147.45–176.09	0.60	0.58–0.63	Early failure

### 3.5 Stratified analyses

**Figure 4:**
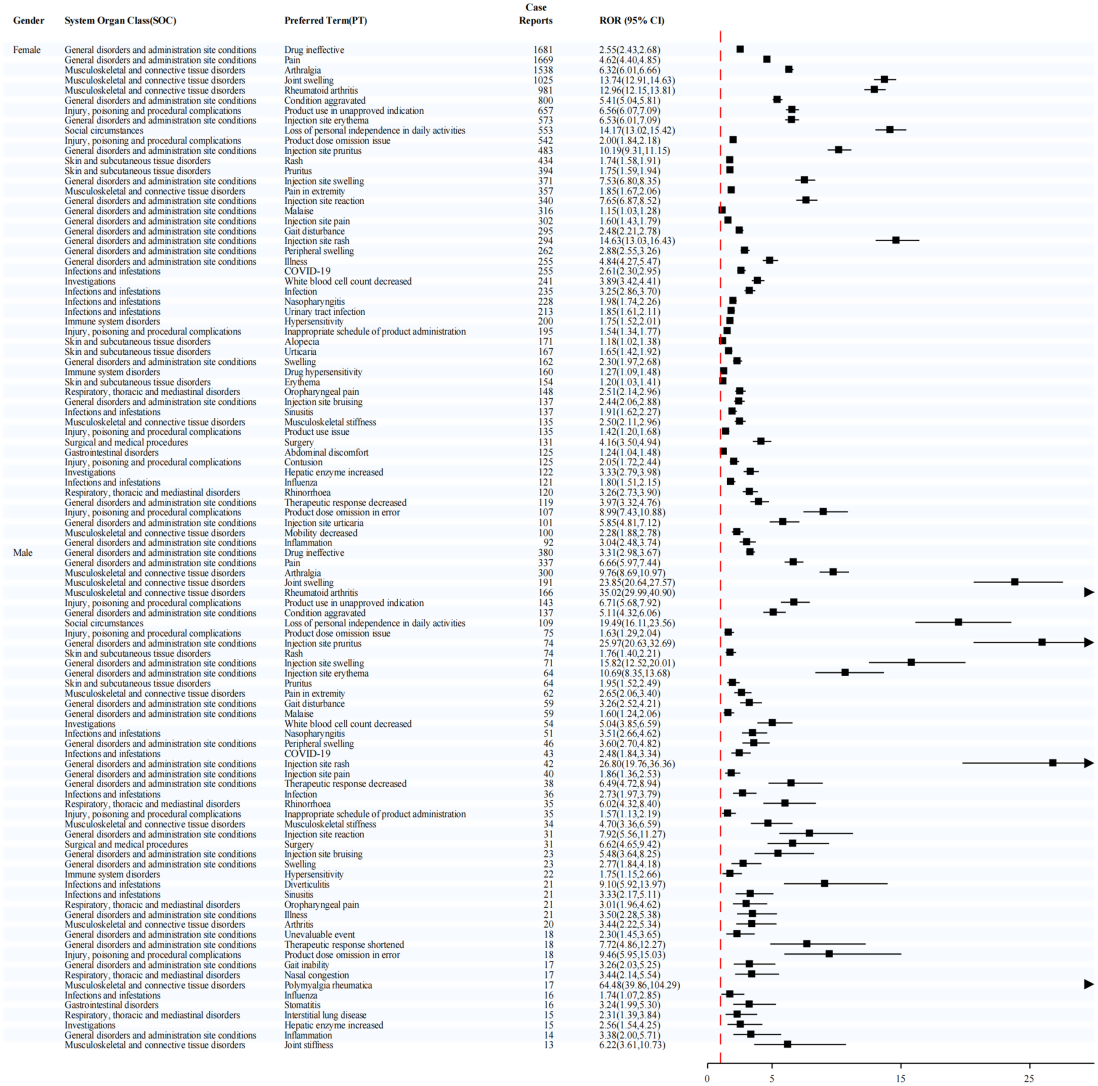
Stratified Disproportionality Analysis of PTs by Sex.

Sex-stratified analysis (Figure 4) revealed notably strong signals for injection site rash in females (ROR = 14.63) and polymyalgia rheumatica in males (ROR = 64.48), suggesting potential sex-specific susceptibility to certain adverse events.

**Figure 5:**
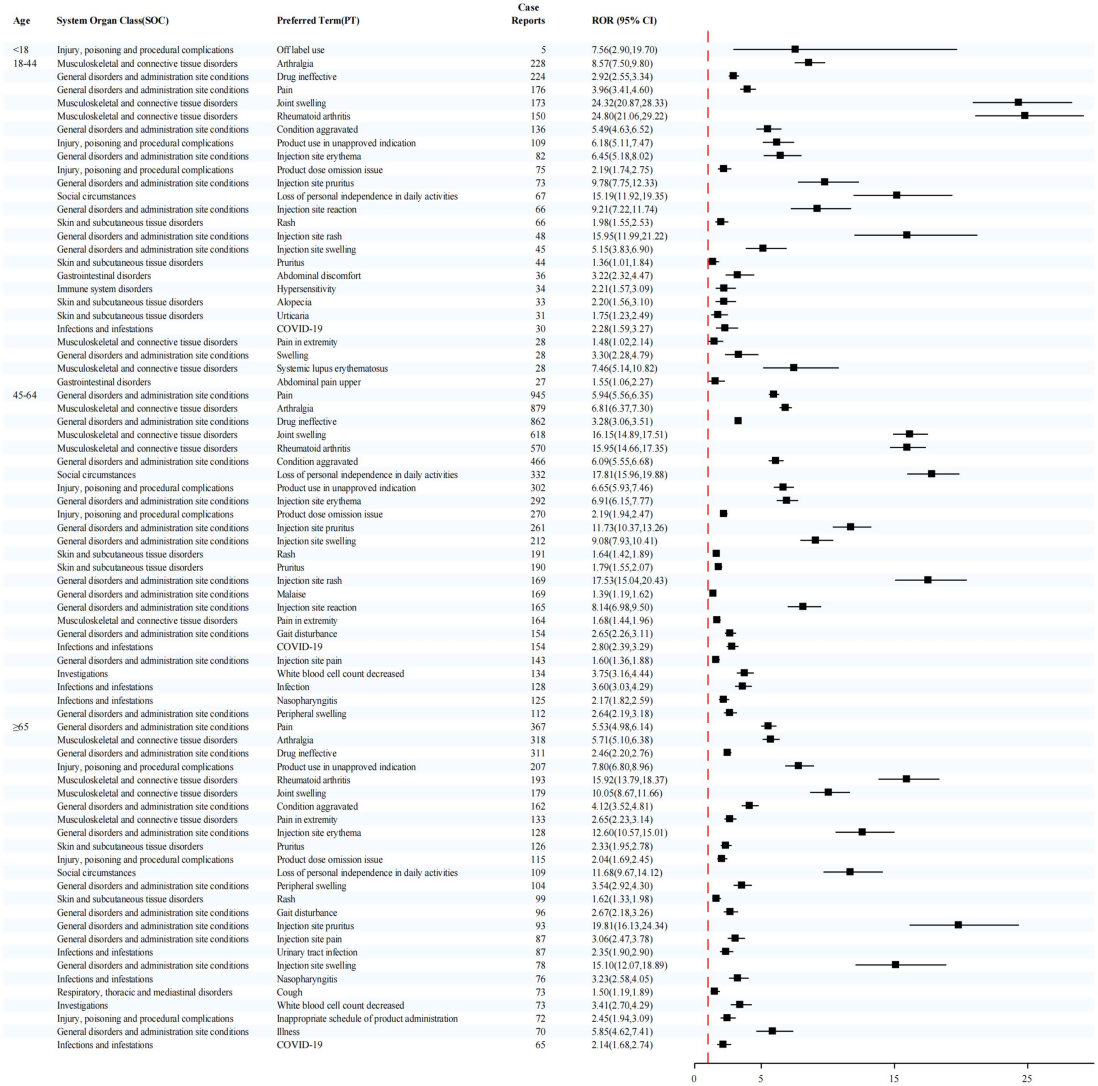
Stratified disproportionality analysis of PTs by age group.

In the age-stratified analysis (Figure 5), the ≥65-year group showed elevated signals for injection site pruritus (ROR = 19.81) and rheumatoid arthritis (ROR = 15.92). In the 45–64 year group, the most prominent signals were loss of personal independence in daily activities (ROR = 17.81) and injection site rash (ROR = 17.53). For the 18–44 year group, the highest signal was also observed for rheumatoid arthritis (ROR = 24.80).

**Figure 6:**
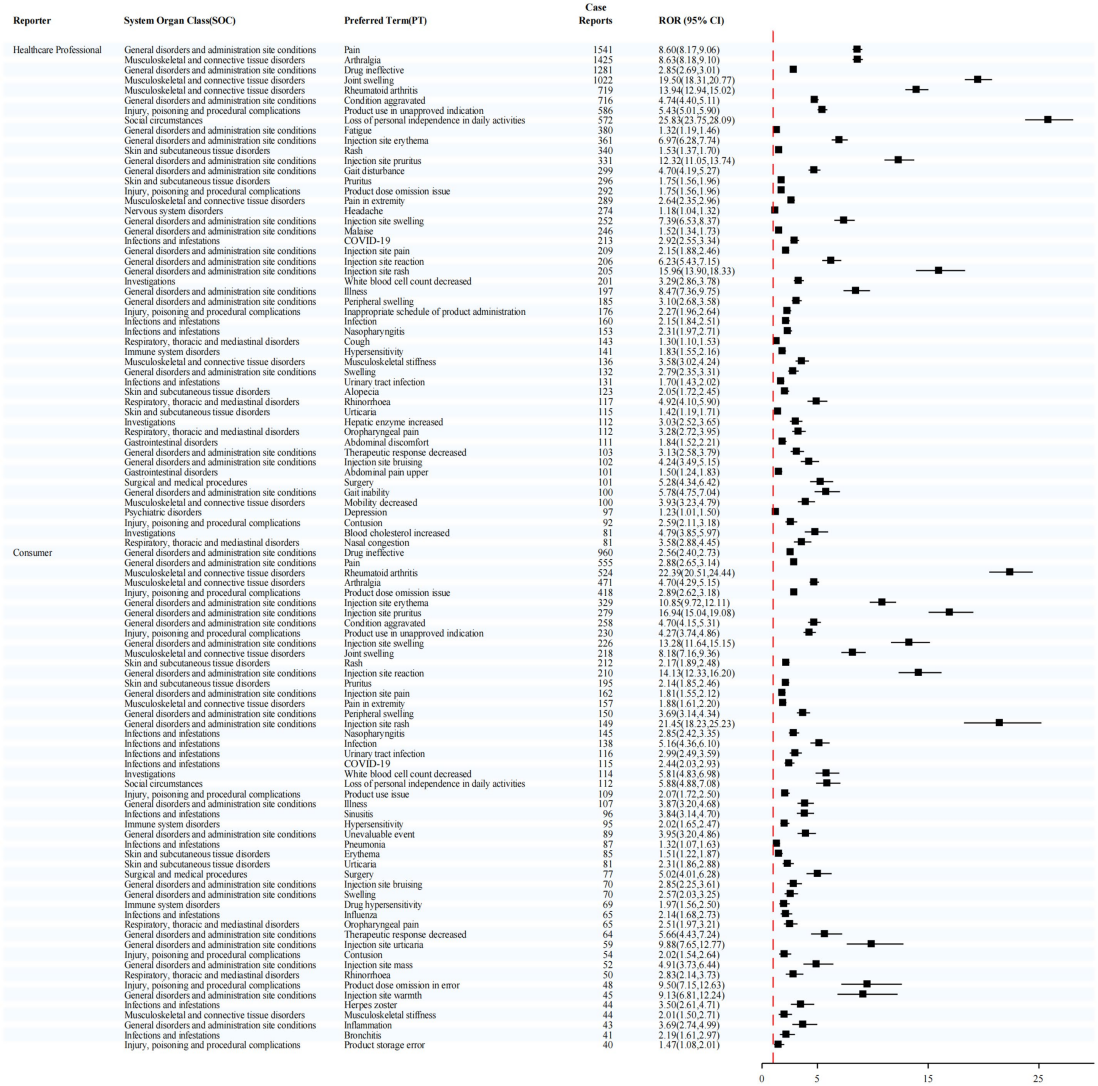
Stratified disproportionality analysis of PTs by reporter type.

Stratification by reporter type (Figure 6) further demonstrated distinct patterns. Among reports submitted by healthcare professionals, the most frequently reported adverse event was pain (1,541 cases), while the strongest signal was for joint swelling (ROR = 19.50). In contrast, among reports submitted by consumers, the most commonly reported event was drug ineffective (960 cases), whereas injection site rash exhibited the highest signal strength (ROR = 21.45).

These findings highlight that the risk of Sarilumab-related adverse events varies across sex, age groups, and reporter types. Such differences underscore the necessity of individualized risk assessment and vigilant pharmacovigilance in clinical practice.

### 3.6 DDIs analysis

**Figure 7:**
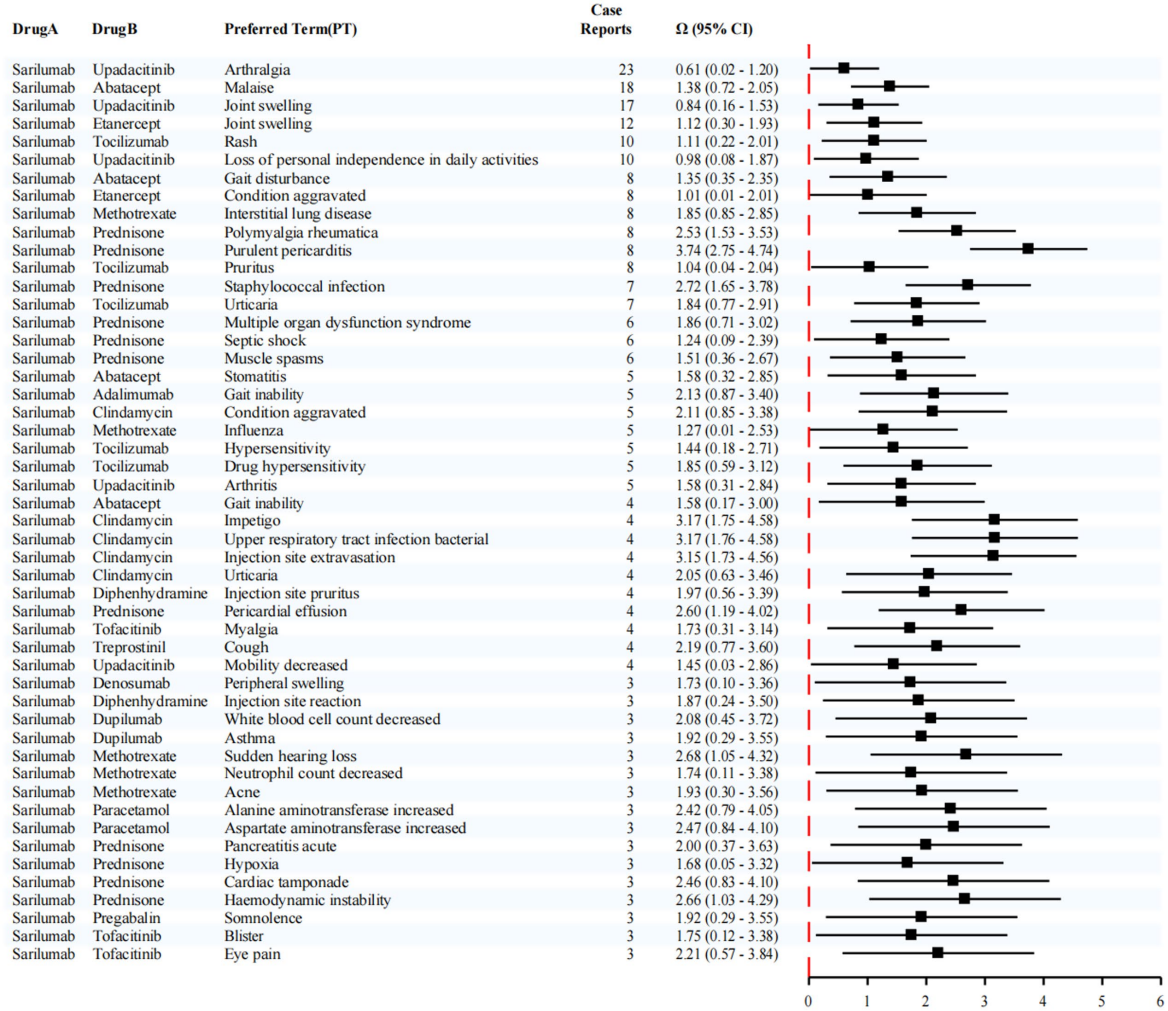
Disproportionality analysis for DDIs with sarilumab. Numbers next to each pair indicate co-report counts (*N*_11_). Pairs with small counts are exploratory only.

This study employed a dedicated disproportionality analysis approach to systematically evaluate potential drug–drug interactions (DDIs) involving Sarilumab. The analytical results are presented in Figure 7. According to established criteria, an interaction signal was considered statistically significant if the lower bound of the 95% confidence interval (CI) for the shrinkage-adjusted odds ratio (*Ω*) exceeded zero.

Notably, the most frequently reported adverse event associated with the co-administration of Sarilumab and Upadacitinib was arthralgia, with 23 cases documented. This combination also produced a statistically significant interaction signal for arthralgia (*Ω* = 0.61, 95% CI: 0.02–1.20). In addition, reports of joint swelling (17 cases, *Ω* = 0.84, 95% CI: 0.16–1.53) and loss of personal independence in daily activities (10 cases, *Ω* = 0.98, 95% CI: 0.08–1.87) were observed. Although these latter signals did not reach statistical significance, they suggest a potential clinical association between combination therapy and lower limb discomfort, which may indirectly compromise functional autonomy and overall quality of life.

Furthermore, in patients receiving Sarilumab in combination with Abatacept, a number of cases involving malaise were reported (18 cases, *Ω* = 1.38, 95% CI: 0.72–2.05), indicating a possible link between this regimen and systemic fatigue or reduced physical function.

Taken together, these findings highlight that Sarilumab may present specific safety concerns when used in combination with other targeted immunosuppressive agents. Enhanced clinical monitoring, individualized risk assessment, and cautious optimization of co-treatment strategies are warranted to ensure treatment safety and improve patient outcomes.

### 3.7 Sensitivity analysis by indication (RA-only)

The overall signal distribution in the RA-only subgroup was highly consistent with the main analysis. At the SOC level, general disorders/administration site conditions and musculoskeletal disorders remained the top two categories, with similar ranking patterns (Supplementary Tables S3–S5). The number of positive PT signals by ROR was comparable between RA-only and overall analyses (249 vs. 218, respectively), and the most frequent PTs largely overlapped (e.g., arthralgia, pain, joint swelling, injection-site reactions, “drug ineffective”).

A difference was observed in the median time-to-onset (77 days in RA-only vs. 31 days overall), suggesting relatively later onset in the RA-only subgroup, although an early-onset risk pattern (*β* < 1 in Weibull analysis) was evident in both. Overall, restricting the analysis to RA cases did not materially change the key safety profile, supporting the robustness of the main findings.

## 4 Discussion

This study systematically reviewed and quantitatively analyzed adverse events (AEs) of Sarilumab using the FAERS database to construct its safety profile in rheumatoid arthritis (RA) treatment. Common label-listed AEs identified included pain, joint swelling, injection site reactions, leukopenia, nausea, and increased infection risk, while severe events involved serious infections, anaphylaxis, gastrointestinal perforation, and hepatotoxicity. Several unlisted AEs, such as fatigue and alopecia, were also observed. These findings underscore the need for close and timely patient monitoring, particularly during early treatment, with long-term follow-up when feasible. Further research is warranted to clarify the mechanisms and risk factors underlying these events.

Previous FAERS-based studies on Sarilumab mainly focused on specific outcomes, such as agranulocytosis/infections (16), general AE profiles without stratified analyses (7), drug-induced liver injury (17), or gastrointestinal perforations (18). In contrast, our study spans a longer period (2017Q2–2025Q1), evaluates all 26 SOCs with multiple algorithms, performs sex/age/reporter-type stratifications, assesses drug–drug interactions, and identifies unlabelled AEs with both early-onset and long-term risk patterns, thus providing a more comprehensive safety profile.

A large body of current research confirms that IL-6 plays a core role in the pathogenesis of RA, participating in major pathological processes such as inflammation, synovial hyperplasia in rheumatoid joints, and cartilage and bone destruction. As a novel IL-6 receptor antagonist, Sarilumab inhibits the activation of the downstream IL-6/STAT3 signaling pathway by binding to IL-6Rα with high affinity, exerting anti-inflammatory, immunomodulatory effects, and slowing synovitis and joint destruction. It typically features clear targeting, long-lasting efficacy, and rapid onset, demonstrating good efficacy in multiple key clinical trials for patients with active RA (19–21). However, its broad immunomodulatory effects may also interfere with homeostasis in multiple systems, triggering a series of adverse reactions that require clinical attention (22).

Although Sarilumab shows promising efficacy in RA, a small proportion of patients may experience cardiovascular adverse events, including QT interval prolongation, hypotension, bradycardia, and arrhythmia (23, 24).; While infrequent, these risks may be higher in individuals with pre-existing vascular disease. Potential mechanisms include: (1) loss of IL-6–mediated myocardial protection via JAK/STAT3 activation, which may reduce resistance to ischemic injury (24); (2) altered autonomic regulation, potentially inducing bradycardia or rhythm disturbances (25); and (3) changes in potassium and calcium channel expression, prolonging action potential duration and QT interval, with a theoretical risk of torsades de pointes (26). These cardiovascular findings in FAERS are consistent with previous reports, underscoring the need for early recognition and timely intervention.

Previous studies have shown that Sarilumab may cause central nervous system (CNS)-related adverse events, such as headache, dizziness, altered consciousness, memory impairment, seizures, and transient loss of consciousness, consistent with our findings (7, 27). Potential mechanisms include: (1) disruption of the excitatory–inhibitory balance in neural circuits due to IL-6 pathway blockade (28, 29); (2) reduced blood–brain barrier stability, allowing harmful substances to enter the CNS and trigger neuroinflammation (30); and (3) elevated levels of excitatory neurotransmitters (e.g., glutamate), inducing epileptiform discharges (31). Therefore, patients with a history of brain injury, epilepsy, or cerebrovascular disease should undergo thorough risk assessment prior to treatment, with close neurological monitoring during therapy.

Compared with other IL-6R monoclonal antibodies such as Tocilizumab, Sarilumab exhibits certain unique pharmacological characteristics. For example, its higher affinity for IL-6R and more persistent inhibitory efficacy may explain its more pronounced trend of neutrophil decline (32). Additionally, when combined with traditional DMARDs, its bioavailability and immunosuppressive intensity may have an additive effect, thereby increasing the complexity of adverse reactions (33). We found that among nearly 15,000 reports, the most common adverse events caused by Sarilumab included injection site reactions, joint pain, leukopenia, liver function abnormalities, and increased infection risk (21, 34). These results are consistent with known mechanisms: IL-6 inhibition can suppress neutrophil maturation, migration, and phagocytic function, thereby weakening the body’s ability to resist pathogens (27, 28). Furthermore, Sarilumab affects hepatocyte lipid metabolism and enzyme expression, potentially leading to elevated ALT/AST and even inducing drug-induced liver injury (20). Therefore, blood routine and liver function tests should be regularly monitored during treatment to promptly identify high-risk individuals (34).

This study conducted a temporal analysis of Sarilumab-related AEs. The results showed that the occurrence of AEs was time-dependent, with nearly half of the events occurring within 30 days of drug use, presenting a clear early-onset risk (35). Additionally, over 15% of AEs occurred after 1 year, suggesting that the drug may also cause long-term potential changes in immunity and organ homeostasis (36). These findings highlight the importance of providing regular monitoring and indicate the need to establish an effective tracking schedule to follow up on drug-related adverse reactions, emphasizing that early monitoring is particularly important for patients taking Sarilumab. Therefore, for long-term users, a systematic follow-up plan should be established, especially for elderly populations with immunosuppression, chronic infection carriers, and patients with liver and kidney dysfunction, who should undergo regular evaluation of systemic organ function (37).

When using Sarilumab, clinicians should comprehensively consider the following key points based on its pharmacological properties, adverse reaction profile, and individual patient differences to minimize potential risks and ensure optimal efficacy and safety: First, indications and contraindications should be fully evaluated. Sarilumab is primarily indicated for patients with moderately to severely active RA, especially those who have had an inadequate response to MTX or TNF inhibitors. It should be avoided or used with caution in patients with severe arrhythmias, a history of epilepsy, or a history of cerebrovascular events; Second, baseline examinations before treatment should include, but are not limited to, blood tests, infection screening, electrocardiogram (ECG), and liver and kidney function tests; Third, dynamic monitoring during treatment should include regular checks of blood routine, liver enzymes, lipids, and infection indicators every 4–8 weeks; Fourth, rational combined medication should avoid drug interactions, and caution should be exercised when co-administering with statins, antihistamines, and potent immunosuppressants. Additionally, clinicians should continuously pay attention to the latest literature and guidelines on Sarilumab use. Through a complete closed-loop management strategy of “pre-assessment—mid-monitoring—post-follow-up,” the risk of severe adverse events can be significantly reduced, improving the safety of RA patients after medication.

In addition, the RA-only sensitivity analysis produced results that were highly consistent with those of the primary analysis, showing only minor variations in the number of detected signals and a longer median time-to-onset (77 days vs. 31 days overall). This concordance further substantiates the robustness of our findings and supports their applicability to the target RA patient population. The observed difference in onset timing may reflect variations in case accrual or reporting patterns between RA-specific and broader patient cohorts.

This study also has certain limitations. First, as a spontaneous reporting system involving physicians, pharmacists, and patients, the FAERS database has inherent shortcomings, including missing information, duplicate reports, and subjective judgments, which may affect data authenticity (8, 38). In addition, to balance sensitivity and specificity, signals detected by only one disproportionality method (ROR, PRR, BCPNN, or MGPS) were interpreted with caution and validated against clinical plausibility, targeted literature verification, and stratified analyses, which may reduce false positives while preserving sensitivity for rare events (9, 11). To ensure the reliability of the results, we applied rigorous data-cleaning procedures, including removing duplicate records, correcting obvious entry errors, and excluding incomplete or unreliable reports, thereby minimizing potential interference from data noise. The large sample size of this study may also help mitigate the impact of inaccuracies in individual reports. Second, the inability to obtain key information such as drug dosage, treatment duration, and concomitant medications limits the assessment of dose–response relationships. Third, the inability to clearly differentiate risks across various indications highlights the need for stratified analyses in future research. Fourth, as most FAERS reports originate from the United States, regional biases may influence reporting patterns. Fifth, given the limited dataset and the small number of reports for some drug combinations, these signals should be considered hypothesis-generating rather than confirmatory. Expanding the sample size and continuously updating the database are essential for a more comprehensive evaluation of Sarilumab’s clinical safety profile. Furthermore, the FAERS database is better suited for identifying potential novel safety signals than for providing precise epidemiological estimates. Therefore, future research should integrate prospective cohort studies and mechanistic investigations to refine the risk characterization of Sarilumab (8, 39).

With the widespread use of biological agents, RA treatment has entered an era of personalized precision medicine (40). A large number of studies have shown that personalized treatment will be an important development direction in the clinical application of Sarilumab. Existing research has explored the correlation between biomarkers such as IL-6, CRP, SAA, and STAT3 and adverse reactions, providing a reference for predicting AE risks (41, 42). Additionally, genetic factors such as HLA loci, FcγR receptor polymorphisms, and metabolic enzyme activity have been confirmed to participate in the pharmacokinetic differences of IL-6 monoclonal antibodies (35, 43). By constructing a genetic-clinical dual-factor risk model, it will help provide patients with precise dose regulation and follow-up pathways.

In the context of coexisting multi-pathway targeted drugs, how to balance efficacy and safety has become the core of clinical decision-making. Based on the report data in the FAERS database, this study revealed potential risk signals during Sarilumab use, providing important evidence for clinicians in pre-medication evaluation, intra-treatment monitoring, and adverse reaction management.

## 5 Conclusion

This study systematically analyzed the adverse events of Sarilumab in the treatment of rheumatoid arthritis based on the FAERS database, revealing that while the drug exerts immunosuppressive efficacy, it may induce common adverse events such as infections, elevated liver enzymes, and leukopenia, as well as potential cardiovascular and neurological risks. As a high-affinity IL-6R antagonist, Sarilumab has advantages such as rapid onset and long-lasting efficacy, but its clinical application should be based on comprehensive patient evaluation and dynamic monitoring. Individualized medication, biomarker prediction, risk population management, and drug interaction assessment are key links determining the safety of Sarilumab use. In the future, based on multi-center prospective studies and multimodal data integration, further improvement of its systemic safety profile and exploration of more precise medication strategies will promote the development of rheumatoid arthritis treatment toward a more efficient and low-risk direction.

## Data Availability

The datasets presented in this study can be found in online repositories. The names of the repository/repositories and accession number(s) can be found in the article/Supplementary material.

## Supplementary material

SUPPLEMENTARY TABLE S1 Two-by-two contingency table for disproportionality.

SUPPLEMENTARY TABLE S2 Five major algorithms used for signal detection.

SUPPLEMENTARY TABLE S3 Signal strength of ADEs at the System Organ Class (SOC) level in the RA-only subgroup (FAERS database).

SUPPLEMENTARY TABLE S4 Top 50 most frequent AEs for Sarilumab in the RA-only subgroup at the preferred term (PT) level.

SUPPLEMENTARY TABLE S5 Time-to-onset analysis in the RA-only subgroup using the Weibull distribution test.

## Glossary

RA: Rheumatoid Arthritis
FAERS: FDA Adverse Event Reporting System
ADE: Adverse Drug Event
AE: Adverse Event
SOC: System Organ Class
ROR: Reporting Odds Ratio
PRR: Proportional Reporting Ratio
BCPNN: Bayesian Confidence Propagation Neural Network
MGPS: Multivariate Gamma-Poisson Shrinker
IL-6: Interleukin-6
IgG1: Immunoglobulin G1
FDA: Food and Drug Administration
DMARDs: Disease-Modifying Anti-Rheumatic Drugs
PT: Preferred Term
MHRA: Medicines and Healthcare products Regulatory Agency
STAT3: Signal Transducer and Activator of Transcription 3
TLOC: Transient Loss of Consciousness
ECG: Electrocardiogram
ALT/AST: Alanine Transaminase / Aspartate Transaminase
CRP: C-Reactive Protein
SAA: Serum Amyloid A
HLA: Human Leukocyte Antigen
FcγR: Fc Gamma Receptor
CSC: China Scholarship Council
