# Supplementary material for: Safety of Sarilumab in the treatment of rheumatoid arthritis: a real-world study based on the FAERS database
Source: Front Med (Lausanne). 2025 Sep 8;12:1665293. doi: 10.3389/fmed.2025.1665293 (PMC12450943; doi:10.3389/fmed.2025.1665293)
Supplement: SUPPLEMENTARY TABLE S1 — Two-by-two contingency table for disproportionality. [file Supplementary_file_1.docx]

**Supplementary Table S1:** Two-by-two contingency table for disproportionality

Analyses

|  | **Target AEs** | **Other AEs** | **Total** |
| --- | --- | --- | --- |
| Sarilumab | a | b | a+b |
| Other drugs | c | d | c+d |
| Total | a+c | b+d | N=a+b+c+d |

Abbreviations: AEs, adverse events; a, the number of reports containing target AEs caused by Sarilumab; b, the number of reports containing other AEs caused by Sarilumab; c, the number of reports containing target AEs caused by other drugs; b, the number of reports containing other AEs caused by other drugs.
