# Supplementary material for: Safety of Sarilumab in the treatment of rheumatoid arthritis: a real-world study based on the FAERS database
Source: Front Med (Lausanne). 2025 Sep 8;12:1665293. doi: 10.3389/fmed.2025.1665293 (PMC12450943; doi:10.3389/fmed.2025.1665293)
Supplement: SUPPLEMENTARY TABLE S2 — Five major algorithms used for signal detection. [file Supplementary_file_2.docx]

**Supplementary Table S2**: Five major algorithms used for signal detection.

| Sarilumab  N | Equation | Criteria |
| --- | --- | --- |
| ROR | 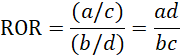  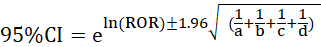 | N≥3 and the 95% CI lower limit>1 |
| PRR | 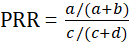  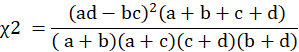 | PRR (95% CI): N≥3 and the 95% CI lower limit>1 PRR (χ2): N≥3, PRR≥2 and χ2≥4 |
| BCPNN | IC=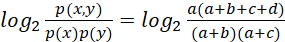  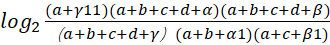  E(IC)=  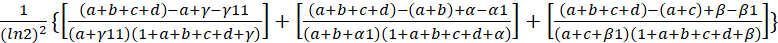  V(IC)=  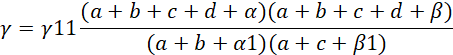  *IC-2SD=E(IC)-2*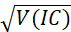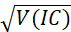  α1=β1, α=β=2 | Lower limit of IC025>0 |
| MGPS | 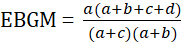  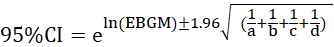 | EBGM05>2 |
|  |  |  |

Abbreviations: AEs, adverse events; a, the number of reports containing target AEs caused by Sarilumab; b, the number of reports containing other AEs caused by Sarilumab; c, the number of reports containing target AEs caused by other drugs; b, the number of reports containing other AEs caused by other drugs; CI, confidence interval; N, the number of reports; χ2, chi-squared; IC, information component; IC025, the lower limit of 95% CI of the IC; E(IC), the IC expectations; V(IC), the variance of IC; EBGM, empirical Bayesian geometric mean; EBGM05, the lower limit of 95% CI of EBGM.
