# Supplementary material for: Safety of Sarilumab in the treatment of rheumatoid arthritis: a real-world study based on the FAERS database
Source: Front Med (Lausanne). 2025 Sep 8;12:1665293. doi: 10.3389/fmed.2025.1665293 (PMC12450943; doi:10.3389/fmed.2025.1665293)
Supplement: SUPPLEMENTARY TABLE S3 — Signal strength of ADEs at the System Organ Class (SOC) level in the RA-only subgroup (FAERS database). [file Supplementary_file_3.docx]

**Supplementary Table S3**

| **System Organ Class(SOC)** | **SOC Code** | **Case reports** | **ROR(95% CI)** | **PRR(95% CI)** | **Chi_Square** | **IC(IC025)** | **EBGM(EBGM05)** |
| --- | --- | --- | --- | --- | --- | --- | --- |
| General disorders and administration site conditions | 10018065 | 10379 | 2.26(2.21,2.31) | 1.85(1.82,1.88) | 4920.33 | 0.89(0.86) | 1.85(1.81) |
| Musculoskeletal and connective tissue disorders | 10028395 | 5987 | 4.22(4.10,4.34) | 3.62(3.54,3.70) | 11948.4 | 1.85(1.81) | 3.62(3.52) |
| Injury, poisoning and procedural complications | 10022117 | 2390 | 0.68(0.66,0.71) | 0.71(0.68,0.73) | 324.98 | -0.50(-0.56) | 0.71(0.68) |
| Infections and infestations | 10021881 | 2364 | 1.43(1.37,1.49) | 1.40(1.34,1.45) | 280.66 | 0.48(0.42) | 1.40(1.34) |
| Skin and subcutaneous tissue disorders | 10040785 | 1938 | 1.12(1.07,1.17) | 1.11(1.06,1.16) | 22.85 | 0.15(0.08) | 1.11(1.06) |
| Gastrointestinal disorders | 10017947 | 1640 | 0.58(0.55,0.61) | 0.60(0.57,0.63) | 475.45 | -0.73(-0.81) | 0.60(0.57) |
| Investigations | 10022891 | 1252 | 0.62(0.59,0.66) | 0.64(0.60,0.67) | 274.55 | -0.65(-0.73) | 0.64(0.60) |
| Nervous system disorders | 10029205 | 1171 | 0.41(0.39,0.43) | 0.43(0.41,0.46) | 957.73 | -1.21(-1.30) | 0.43(0.41) |
| Respiratory, thoracic and mediastinal disorders | 10038738 | 1137 | 0.74(0.70,0.79) | 0.75(0.71,0.80) | 97.68 | -0.41(-0.50) | 0.75(0.71) |
| Social circumstances | 10041244 | 736 | 4.99(4.63,5.36) | 4.89(4.56,5.26) | 2284.74 | 2.29(2.17) | 4.88(4.54) |
| Surgical and medical procedures | 10042613 | 550 | 1.26(1.15,1.37) | 1.25(1.15,1.36) | 28.20 | 0.32(0.20) | 1.25(1.15) |
| Psychiatric disorders | 10037175 | 512 | 0.27(0.25,0.30) | 0.28(0.26,0.31) | 973.40 | -1.81(-1.94) | 0.28(0.26) |
| Immune system disorders | 10021428 | 390 | 1.10(0.99,1.21) | 1.10(0.99,1.21) | 3.46 | 0.14(-0.01) | 1.10(0.99) |
| Cardiac disorders | 10007541 | 226 | 0.26(0.23,0.30) | 0.27(0.24,0.31) | 458.40 | -1.89(-2.08) | 0.27(0.24) |
| Vascular disorders | 10047065 | 222 | 0.32(0.28,0.37) | 0.32(0.28,0.37) | 318.09 | -1.62(-1.81) | 0.33(0.28) |
| Metabolism and nutrition disorders | 10027433 | 218 | 0.31(0.27,0.35) | 0.31(0.27,0.36) | 334.94 | -1.67(-1.86) | 0.31(0.27) |
| Blood and lymphatic system disorders | 10005329 | 199 | 0.36(0.31,0.42) | 0.37(0.32,0.42) | 222.29 | -1.45(-1.65) | 0.37(0.32) |
| Eye disorders | 10015919 | 199 | 0.31(0.27,0.35) | 0.31(0.27,0.36) | 312.31 | -1.69(-1.89) | 0.31(0.27) |
| Neoplasms benign, malignant and unspecified (incl cysts and polyps) | 10029104 | 175 | 0.21(0.18,0.24) | 0.21(0.18,0.24) | 535.57 | -2.25(-2.47) | 0.21(0.18) |
| Renal and urinary disorders | 10038359 | 147 | 0.24(0.20,0.28) | 0.24(0.21,0.28) | 355.55 | -2.05(-2.28) | 0.24(0.21) |
| Hepatobiliary disorders | 10019805 | 125 | 0.42(0.35,0.50) | 0.42(0.35,0.50) | 99.98 | -1.24(-1.50) | 0.42(0.35) |
| Ear and labyrinth disorders | 10013993 | 73 | 0.52(0.42,0.66) | 0.52(0.42,0.66) | 31.59 | -0.93(-1.26) | 0.52(0.42) |
| Product issues | 10077536 | 44 | 0.08(0.06,0.11) | 0.08(0.06,0.11) | 457.31 | -3.60(-4.00) | 0.08(0.06) |
| Reproductive system and breast disorders | 10038604 | 35 | 0.12(0.09,0.17) | 0.12(0.09,0.17) | 220.06 | -3.02(-3.46) | 0.12(0.09) |
| Endocrine disorders | 10014698 | 20 | 0.24(0.16,0.38) | 0.24(0.16,0.38) | 47.25 | -2.04(-2.62) | 0.24(0.16) |
| Pregnancy, puerperium and perinatal conditions | 10036585 | 16 | 0.12(0.07,0.19) | 0.12(0.07,0.19) | 106.89 | -3.09(-3.72) | 0.12(0.07) |
| Congenital, familial and genetic disorders | 10010331 | 7 | 0.07(0.03,0.15) | 0.07(0.03,0.15) | 82.53 | -3.77(-4.62) | 0.07(0.03) |

Note:ranked by case reports
