# Supplementary material for: Safety of Sarilumab in the treatment of rheumatoid arthritis: a real-world study based on the FAERS database
Source: Front Med (Lausanne). 2025 Sep 8;12:1665293. doi: 10.3389/fmed.2025.1665293 (PMC12450943; doi:10.3389/fmed.2025.1665293)
Supplement: SUPPLEMENTARY TABLE S4 — Top 50 most frequent AEs for Sarilumab in the RA-only subgroup at the preferred term (PT) level. [file Supplementary_file_4.docx]

**Supplementary Table S4**

| **System Organ Class(SOC)** | **Preferred Term(PT)** | **Case reports** | **ROR (95% CI)** | **PRR (95% CI)** | **Chi Square** | **IC (IC025)** | **EBGM (EBGM05)** |
| --- | --- | --- | --- | --- | --- | --- | --- |
| Musculoskeletal and connective tissue disorders | Arthralgia | 1792 | 8.92  (8.51,9.36) | 8.48  (8.11,8.87) | 11851.0 | 3.08  (3.00) | 8.45  (8.05) |
| General disorders and administration site conditions | Drug ineffective | 1762 | 2.68  (2.55,2.81) | 2.59  (2.47,2.71) | 1750.38 | 1.37  (1.30) | 2.58  (2.46) |
| General disorders and administration site conditions | Pain | 1709 | 5.57  (5.30,5.84) | 5.32  (5.08,5.58) | 6043.93 | 2.41  (2.33) | 5.31  (5.06) |
| Musculoskeletal and connective tissue disorders | Joint swelling | 1263 | 21.88  (20.68,23.15) | 21.06  (19.95,22.24) | 23895.7 | 4.38  (4.27) | 20.83  (19.68) |
| Musculoskeletal and connective tissue disorders | Rheumatoid arthritis | 1247 | 23.48  (22.18,24.85) | 22.61  (21.40,23.88) | 25473.1 | 4.48  (4.37) | 22.34  (21.10) |
| General disorders and administration site conditions | Condition aggravated | 893 | 6.08  (5.69,6.50) | 5.94  (5.57,6.34) | 3673.58 | 2.57  (2.46) | 5.92  (5.54) |
| Social circumstances | Loss of personal independence in daily activities | 650 | 19.10  (17.66,20.65) | 18.73  (17.35,20.22) | 10806.8 | 4.21  (4.06) | 18.54  (17.15) |
| General disorders and administration site conditions | Injection site erythema | 491 | 7.82  (7.15,8.55) | 7.72  (7.07,8.43) | 2864.35 | 2.94  (2.79) | 7.69  (7.03) |
| General disorders and administration site conditions | Injection site pruritus | 446 | 13.30  (12.11,14.61) | 13.13  (11.97,14.40) | 4967.30 | 3.71  (3.53) | 13.04  (11.87) |
| General disorders and administration site conditions | Injection site swelling | 347 | 9.16  (8.24,10.18) | 9.07  (8.17,10.07) | 2482.36 | 3.17  (2.99) | 9.03  (8.12) |
| General disorders and administration site conditions | Gait disturbance | 336 | 3.30  (2.96,3.67) | 3.27  (2.94,3.64) | 531.65 | 1.71  (1.54) | 3.27  (2.94) |
| General disorders and administration site conditions | Injection site reaction | 282 | 8.08  (7.19,9.09) | 8.02  (7.14,9.01) | 1726.78 | 3.00  (2.79) | 7.99  (7.10) |
| General disorders and administration site conditions | Peripheral swelling | 262 | 3.39  (3.00,3.83) | 3.37  (2.99,3.81) | 437.73 | 1.75  (1.56) | 3.37  (2.98) |
| General disorders and administration site conditions | Illness | 259 | 5.63  (4.98,6.36) | 5.59  (4.95,6.31) | 974.82 | 2.48  (2.27) | 5.58  (4.93) |
| General disorders and administration site conditions | Injection site rash | 256 | 17.09  (15.11,19.34) | 16.97  (15.01,19.18) | 3812.00 | 4.07  (3.80) | 16.82  (14.86) |
| Infections and infestations | COVID-19 | 248 | 2.66  (2.35,3.02) | 2.65  (2.34,3.00) | 254.84 | 1.40  (1.21) | 2.65  (2.34) |
| Infections and infestations | Infection | 237 | 3.26  (2.87,3.70) | 3.24  (2.85,3.68) | 367.44 | 1.69  (1.49) | 3.24  (2.85) |
| Investigations | White blood cell count decreased | 211 | 3.74  (3.27,4.29) | 3.73  (3.26,4.26) | 420.82 | 1.90  (1.68) | 3.72  (3.25) |
| General disorders and administration site conditions | Swelling | 157 | 2.78  (2.38,3.25) | 2.77  (2.37,3.24) | 177.98 | 1.47  (1.22) | 2.77  (2.37) |
| Surgical and medical procedures | Surgery | 135 | 4.79  (4.04,5.67) | 4.77  (4.03,5.65) | 402.13 | 2.25  (1.96) | 4.76  (4.02) |
| General disorders and administration site conditions | Therapeutic response decreased | 133 | 4.33  (3.65,5.13) | 4.31  (3.64,5.11) | 337.98 | 2.11  (1.82) | 4.31  (3.63) |
| Musculoskeletal and connective tissue disorders | Musculoskeletal stiffness | 122 | 2.71  (2.27,3.23) | 2.70  (2.26,3.22) | 130.68 | 1.43  (1.15) | 2.70  (2.26) |
| Respiratory, thoracic and mediastinal disorders | Oropharyngeal pain | 116 | 2.42  (2.01,2.90) | 2.41  (2.01,2.89) | 95.93 | 1.27  (0.98) | 2.41  (2.01) |
| General disorders and administration site conditions | Injection site bruising | 115 | 2.90  (2.41,3.48) | 2.89  (2.41,3.47) | 142.24 | 1.53  (1.24) | 2.89  (2.40) |
| Injury, poisoning and procedural complications | Product dose omission in error | 109 | 9.61  (7.96,11.61) | 9.58  (7.94,11.57) | 833.86 | 3.25  (2.87) | 9.54  (7.90) |
| Respiratory, thoracic and mediastinal disorders | Rhinorrhoea | 108 | 3.28  (2.72,3.97) | 3.28  (2.71,3.96) | 170.75 | 1.71  (1.40) | 3.27  (2.71) |
| Investigations | Hepatic enzyme increased | 102 | 3.03  (2.49,3.68) | 3.02  (2.49,3.67) | 138.04 | 1.59  (1.28) | 3.02  (2.49) |
| Musculoskeletal and connective tissue disorders | Mobility decreased | 102 | 2.81  (2.31,3.41) | 2.80  (2.31,3.40) | 118.14 | 1.48  (1.17) | 2.80  (2.30) |
| General disorders and administration site conditions | Gait inability | 98 | 3.27  (2.68,3.99) | 3.27  (2.68,3.98) | 153.98 | 1.71  (1.38) | 3.26  (2.68) |
| General disorders and administration site conditions | Inflammation | 92 | 3.56  (2.90,4.37) | 3.56  (2.90,4.36) | 168.84 | 1.83  (1.49) | 3.55  (2.89) |
| General disorders and administration site conditions | Injection site urticaria | 86 | 7.07  (5.72,8.74) | 7.06  (5.71,8.72) | 445.46 | 2.81  (2.41) | 7.03  (5.69) |
| Gastrointestinal disorders | Stomatitis | 82 | 2.66  (2.14,3.30) | 2.65  (2.14,3.29) | 84.42 | 1.41  (1.06) | 2.65  (2.13) |
| Respiratory, thoracic and mediastinal disorders | Nasal congestion | 80 | 2.69  (2.16,3.35) | 2.68  (2.16,3.34) | 84.50 | 1.42  (1.07) | 2.68  (2.15) |
| Infections and infestations | Herpes zoster | 76 | 2.54  (2.03,3.18) | 2.54  (2.03,3.17) | 70.66 | 1.34  (0.98) | 2.53  (2.02) |
| Investigations | Blood cholesterol increased | 73 | 3.05  (2.43,3.84) | 3.05  (2.42,3.83) | 100.30 | 1.61  (1.23) | 3.04  (2.42) |
| General disorders and administration site conditions | Injection site warmth | 71 | 6.51  (5.16,8.22) | 6.50  (5.15,8.20) | 329.38 | 2.70  (2.25) | 6.48  (5.13) |
| General disorders and administration site conditions | Injection site mass | 68 | 3.60  (2.84,4.57) | 3.59  (2.83,4.56) | 127.12 | 1.84  (1.44) | 3.59  (2.83) |
| Musculoskeletal and connective tissue disorders | Systemic lupus erythematosus | 63 | 3.96  (3.09,5.07) | 3.95  (3.09,5.06) | 138.68 | 1.98  (1.55) | 3.95  (3.08) |
| Injury, poisoning and procedural complications | Intentional dose omission | 49 | 4.09  (3.09,5.41) | 4.09  (3.09,5.41) | 113.95 | 2.03  (1.53) | 4.08  (3.08) |
| Investigations | Swollen joint count increased | 48 | 41.70  (31.32,55.53) | 41.64  (31.28,55.43) | 1860.46 | 5.35  (4.07) | 40.71  (30.57) |
| Cardiac disorders | Pericarditis | 46 | 6.27  (4.69,8.37) | 6.26  (4.69,8.36) | 202.57 | 2.64  (2.07) | 6.24  (4.67) |
| Musculoskeletal and connective tissue disorders | Joint stiffness | 46 | 3.40  (2.55,4.54) | 3.40  (2.55,4.54) | 77.78 | 1.76  (1.27) | 3.39  (2.54) |
| Surgical and medical procedures | Knee arthroplasty | 43 | 4.24  (3.14,5.72) | 4.23  (3.14,5.71) | 105.95 | 2.08  (1.54) | 4.23  (3.13) |
| Gastrointestinal disorders | Duodenal ulcer perforation | 42 | 14.89  (10.98,20.17) | 14.87  (10.98,20.14) | 538.81 | 3.88  (3.04) | 14.75  (10.89) |
| Injury, poisoning and procedural complications | Contraindicated product administered | 42 | 2.95  (2.18,4.00) | 2.95  (2.18,3.99) | 54.02 | 1.56  (1.05) | 2.95  (2.18) |
| Infections and infestations | Diverticulitis | 41 | 2.91  (2.14,3.95) | 2.90  (2.14,3.94) | 51.13 | 1.54  (1.03) | 2.90  (2.14) |
| Skin and subcutaneous tissue disorders | Pemphigus | 41 | 8.56  (6.29,11.63) | 8.55  (6.29,11.61) | 271.97 | 3.09  (2.41) | 8.51  (6.26) |
| Infections and infestations | Ear infection | 40 | 2.95  (2.16,4.02) | 2.95  (2.16,4.02) | 51.41 | 1.56  (1.04) | 2.94  (2.16) |
| General disorders and administration site conditions | Injection site irritation | 38 | 5.88  (4.28,8.09) | 5.87  (4.27,8.08) | 153.21 | 2.55  (1.92) | 5.86  (4.26) |
| Infections and infestations | Localised infection | 38 | 3.01  (2.19,4.14) | 3.01  (2.19,4.14) | 51.00 | 1.59  (1.05) | 3.01  (2.19) |

Note1:ranked by Reports

Note2:Signals are detected when all the following criteria are met:a ≥ 3, PRR ≥2 and Chi-Square ≥ 4, lower limit of 95% CI of ROR > 1, IC025 > 0, EBGM05 > 2.
