# Supplementary material for: Safety of Sarilumab in the treatment of rheumatoid arthritis: a real-world study based on the FAERS database
Source: Front Med (Lausanne). 2025 Sep 8;12:1665293. doi: 10.3389/fmed.2025.1665293 (PMC12450943; doi:10.3389/fmed.2025.1665293)
Supplement: SUPPLEMENTARY TABLE S5 — Time-to-onset analysis in the RA-only subgroup using the Weibull distribution test. [file Supplementary_file_5.docx]

**Supplementary Table S5**

|  | | **Weibull distribution** | | | |  |
| --- | --- | --- | --- | --- | --- | --- |
| **Cases** | **TTO (days)** | **Scale parameter** | | **Shape parameter** | |  |
| **n** | **median(IQR)** | **α** | **95% CI** | **β** | **95% CI** | **Failure type** |
| 1293 | 77.00(12.00,291.00) | 236.03 | 215.17 - 258.92 | 0.68 | 0.65 - 0.71 | Early failure |
